# Perceptions of Fetal Alcohol Spectrum Disorder (FASD) at a Mental Health Outpatient Treatment Provider in Minnesota

**DOI:** 10.3390/ijerph16010016

**Published:** 2018-12-21

**Authors:** Jerrod Brown, Diane Harr

**Affiliations:** 1 Pathways Counseling Center, Inc., St. Paul, MN 55104, USA; 2 Department of Human Services, College of Humanities and Social Sciences, Concordia University, St. Paul, MN 55104, USA; 3 American Institute for the Advancement of Forensic Studies, St. Paul, MN 55104, USA; 4 Department of Graduate Teacher Education, College of Education, Concordia University, St. Paul, MN 55104, USA; dharr@csp.edu

**Keywords:** fetal alcohol spectrum disorder, education, mental health, training

## Abstract

Resulting from prenatal exposure to alcohol, Fetal Alcohol Spectrum Disorder (FASD) is characterized by deficits in adaptive and cognitive functioning. This disorder is typically accompanied by co-occurring disorders and conditions (e.g., mood, anxiety, psychosis, and substance use disorders). This complicated presentation of diverse symptoms makes the process of screening, assessing, and diagnosing FASD very difficult, limiting the likelihood that clients receive the treatment and services that they need. Although mental health care providers have an opportunity to intervene on behalf of clients with FASD, professionals may not be very familiar or comfortable with this complicated and life-altering disorder. The present study explores the familiarity of 79 mental health outpatient treatment professionals’ personal knowledge and training about FASD. Findings suggest that the majority of respondents had received at least some FASD training, understood the basic symptoms of FASD, and were realistic about FASD’s impact on treatment.

## 1. Introduction

Estimates of Fetal Alcohol Spectrum Disorder’s (FASD) prevalence in the United States range from 3% to 5% [1]. Disorders that fall under the FASD umbrella include fetal alcohol syndrome (FAS), alcohol related neurodevelopmental disorder (ARND), and alcohol related birth defects (ARBD). This irreversible affliction is caused by prenatal exposure to alcohol [2]. FASD is characterized by deficits in cognitive (e.g., executive function, information processing, short- and long-term memory, and attentional control), social (e.g., immaturity and verbal and non-verbal communication skills), and adaptive functioning [3,4,5,6,7]. In a minority of cases, physical impairments like dysmorphia and musculoskeletal conditions are even present [6,8,9,10]. These diverse and ranging symptoms often have devastating consequences on an individual’s functioning at school and work along with independent living skills such as managing a household and financial responsibilities [11,12,13].

In addition to the symptoms of FASD, the disorder is typically accompanied by co-occurring disorders and conditions. In fact, researchers have estimated that nearly 90% of those with FASD also exhibit at least one comorbid condition [14,15]. These co-occurring conditions are often mood (i.e., depression and bipolar), anxiety, psychosis (i.e., schizophrenia), behavioral (e.g., ADHD and conduct disorder), attachment (e.g., reactive), or substance use disorders [15,16,17]. As such, the presentation of FASD symptoms and co-occurring disorders can present in vastly different combinations on a client-by-client basis [15,18,19]. The wide array of symptoms from both FASD and any co-occurring conditions make the process of screening, assessing, and diagnosing FASD very difficult. This contributes to the frequent missed diagnosis and misdiagnosis of the disorder [2,18,20]. A consequence of these difficulties is that those with FASD are less likely to receive the treatment and services that they need [21].

In an effort to improve identification and diagnosis, FASD was included in the Diagnostic and Statistical Manual, Fifth Edition [22]. This is called Neurodevelopmental Disorder Associated with Prenatal Alcohol Exposure (ND-PAE) and is considered a condition in need of further study. Diagnostic criteria include the presence of prenatal alcohol exposure along with impairments in neurocognitive, self-regulation, and adaptive functioning. While these diagnostic criteria undergo clinical and scientific evaluation, FASD must still be diagnosed by medical professionals in the meantime.

When not accurately diagnosed and properly treated and supported, individuals with FASD are prone to negative life outcomes including involvement in the criminal justice system [23,24,25]. For example, Streissguth, Barr, Kogan, and Bookstein [15] estimate that approximately 60% of individuals with FASD enter into the criminal justice system at some point in their lives. This legal involvement could be the direct consequence of the cognitive, social, and adaptive functioning impairments of FASD [26]. Unfortunately, these FASD symptoms also limit an individual’s capacity to function in the criminal justice system. Specifically, individuals with FASD could have difficulty making important legal decisions (e.g., waiving Miranda rights), participating in police interviews and interrogations, and being competent to stand trial [27,28,29,30,31].

Although mental health care providers have an opportunity to intervene on behalf of clients with FASD, professionals may not be very familiar with this devastating disorder [32]. This concern is emphasized by the findings of a survey about psychologists’ knowledge of FASD from 2007 of 447 doctoral-level American Psychological Association members [33]. This questionnaire was designed to gauge a respondent’s knowledge of alcohol’s effects during pregnancy and FASD. The results indicated that these professionals generally had limited familiarity with the prevention, diagnosis, and treatment of prenatal alcohol exposure [33]. Further, a 2003 study of 391 Canadian psychiatrists found approximately half of respondents felt unprepared to assist patients in the management of alcohol misuse and its consequences [34].

The present study, focused specifically on FASD, was developed to explore the generalizability of previous research and any changes over the last decade. Specifically, mental health outpatient treatment providers in a Midwestern state were surveyed about their training and knowledge of FASD. We have three hypotheses. First, we expect that professionals will have had at least some training on FASD. Second, we predict these professionals will have a strong grasp of foundational knowledge in the area of FASD. Third, we anticipate that professionals will believe additional training and the availability of FASD screening tools would be beneficial.

## 2. Materials and Methods

The survey titled Perceptions of Fetal Alcohol Spectrum Disorder (FASD) at a Mental Health Outpatient Treatment Provider in Minnesota was constructed and administered using Google Forms. The mental health outpatient treatment provider sampled for this survey was Headway Emotional Health Services (HEHS). This organization has provided care and services in the Twin Cities Minnesota community for over 40 years. Headway treats children, adolescents, adults, couples, and families across a wide range of circumstances. This organization was selected specifically because it has many years of experience treating individuals with either suspected or confirmed FASD. The anonymous survey was distributed electronically by a representative of Headway to the staff of the organization (*n* = 189). After the initial recruitment email, two subsequent reminder emails were sent out between 20 August 2018, and 20 September 2018, requesting participation. Prior to completing the survey, participants were prompted with an informed consent page. Participants were required to select the “Agree” option to begin the survey. Responses were stored in a Google Sheets document for later analysis. This study was approved by the Institutional Review Board at Concordia University, St. Paul, Minnesota (Study Number: 2017_103).

Of 189 potential participants, 42% (*n* = 79) at least partially completed the survey submission. The respondents were mostly women (82%; *n* = 65) and between the ages of 25–34 (49%, *n* = 32) or 35–44 (33%, *n* = 26). Although all respondents earned at least an undergraduate degree, such as an Associate of Arts (AA), Bachelor of Arts (BA), 65% (*n* = 51) had a graduate degree, including Master of Arts (MA) or other Master’s level degrees, and 6% (*n* = 5) had a doctorate degree. The majority of respondents had 10 years or less of experience in the field of mental health, with 41% (*n* = 32) having less than 5 years of experience and 37% (*n* = 29) having between 5 and 10 years of experience.

The survey consisted of 30 closed-ended response questions and two open-ended long answer questions. Of these 32 items, 25 items were specific to FASD (e.g., familiarity with FASD symptoms, impact of FASD on treatment, and FASD training received) and seven items pertained to demographic information (i.e., age, gender, education history, and job position). Each survey question can be reviewed in Appendix A.

## 3. Results

### 3.1. Training

To explore the level, recency, and quality of FASD training among respondents, a series of questions were asked. First, participants were asked if they had ever received FASD training. Almost two-thirds of participants (62%, *n* = 49) indicated that they had received FASD training in the past, whereas 38% (*n* = 30) had never received FASD.

Second, among those who had received FASD training, participants were asked about how long ago the FASD training took place. For most respondents, the training was between 1 and 5 years ago (61%, *n* = 31). The next most common responses were that the training took place between 5 and 10 years ago (18%, *n* = 9), within the last 12 months (16%, *n* = 8), and more than 10 years go (4%, *n* = 2). Of interest, a participant who indicated never having had a training did select between 1 and 5 years ago.

Third, the participants were asked if FASD training was helpful in determining the presence of FASD. Of those that received training, most found the training “Helpful” (33%, *n* = 26). The other responses were “Somewhat Helpful” (10%, *n* = 8), “Somewhat Unhelpful” (9%, *n* = 7), and “Not Helpful at All” or “Extremely Helpful” (4%, *n* = 3). Nonetheless, a substantial proportion of the group, consistent with above, still had not received FASD training (41%, *n* = 32).

Fourth, the participants were asked if they felt education/training in the identification of FASD should occur regularly. The most common responses were that training should occur every 2 years on a recurring basis (47%, *n* = 37) or every 12 months on a recurring basis (33%, *n* = 26). Fewer respondents endorsed having training every 5 years on a recurring basis (13%, *n* = 10), every 6 months on a recurring basis (6%, *n* = 5), or not having regular training at all (1%, *n* = 1).

Finally, the participants were asked if a continuing education course that addresses the interaction between FASD and the mental health system would be beneficial. The response was overwhelmingly positive. The vast majority responded “Yes” (96%, *n* = 76), whereas only 4% (*n* = 3) responded “No.”

### 3.2. Diagnostic Knowledge

**Figure 1:**
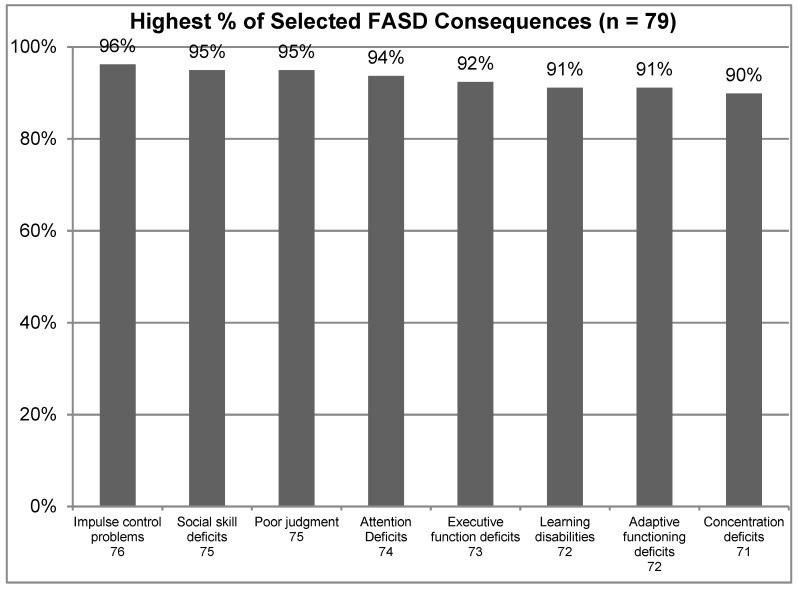
Highest % of selected Fetal Alcohol Spectrum Disorder (FASD) consequences.

To better understand the knowledge of mental health practitioners in the area of FASD, a range of questions were asked about the disorder and its presentation. First, the participants were asked to select all of the consequences associated with FASD from a list of 15 choices, with those selected by 90% or more of all study participants shown in Figure 1 below. This included Impulse Control Problems (96%), Social Skill Deficits (95%), Poor judgment (95%), Attention Deficits (94%), Executive Function Deficits (92%), Learning Disabilities (91%), Adaptive Functioning Deficits (91%), and Concentration Deficits (90%). These findings indicate that the mental health professionals were very familiar with many of the most commonly associated consequences of FASD.

**Figure 2:**
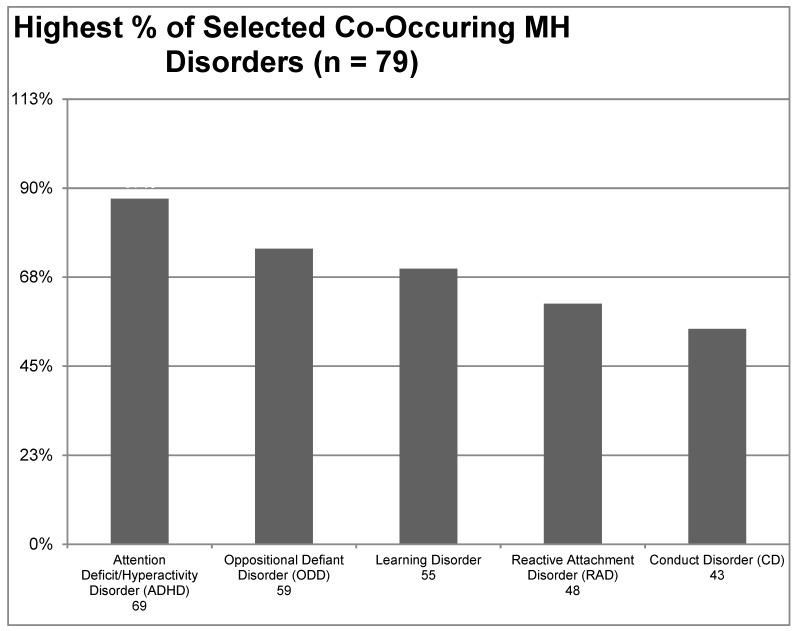
Highest % of selected co-occurring mental health disorders.

Similarly, participants were asked to identify any mental health disorders that commonly co-occur with FASD from a list of 22 choices with those selected by 50% or more of all study participants shown in Figure 2 below. The most common co-occurring mental health disorders were Attention Deficit/Hyperactivity Disorder (87%), Oppositional Defiant Disorder (75%), Learning Disorder (70%), Reactive Attachment Disorder (61%), and Conduct Disorder (54%). Less commonly endorsed disorders included Post-Traumatic Stress Disorder (33%), Autism Spectrum Disorder (28%), Sleep disorders (22%), and Anti-Social Personality Disorder (19%). 

Another important topic of consideration is how often individuals with FASD present with facial abnormalities. This includes physical features like wideset eyes, thin upper lip, smooth philtrum, upturned nose, epicanthal folds, and small head size. The majority of respondents selected that facial features were present in 1% to 25% (53%, *n* = 42). Others responded that facial features were present in 26% to 50% (32%, *n* = 25), 51% to 75% (9%, *n* = 7), or 76% to 100% (6%, *n* = 5) of FASD cases.

In light of these ranging diagnostic features and co-occurring disorders, the participants were asked about the potential utility of a FASD screening tool or application. The overwhelming response was that a screening tool or application would be helpful (97%, *n* = 76). Only 3% (*n* = 2) of respondents did not believe such an instrument would be helpful.

The knowledge of mental health professionals on the impacts of FASD on the various facets of everyday life was also explored. When asked if FASD significantly impacts the social skills of the client, most participants “Agreed” that social skills were impaired (62%, *n* = 49) and a substantial proportion of participants even “Strongly Agreed” (28%, *n* = 22). Fewer participants endorsed the options of “Neither Disagree nor Agree” (8%, *n* = 6) or “Strongly Disagree” (3%, *n* = 2). Similarly, participants were asked if FASD significantly impairs parenting ability. This notion was “Agreed” with by just under half of the participants (42%, *n* = 33). However, others responded “Neither Disagree nor Agree” (33%, *n* = 26), “Strongly Agree” (23%, *n* = 18), “Disagree” (1%, *n* = 1), or “Strongly Disagree” (1%, *n* = 1).

### 3.3. Treatment

To explore the perceptions of mental health professionals on the impact of FASD on treatment, a series of questions were developed and asked. First, the participants were asked if FASD plays a role in patient adherence to a treatment plan. A majority of the participants “Agree” that FASD impacts adherence (57%, *n* = 45). That said, others responded “Neither Disagree nor Agree” (20%, *n* = 16), “Strongly Agree” (13%, *n* = 10), “Disagree” (6%, *n* = 5), or “Strongly Disagree” (4%, *n* = 3). Second, the participants were asked if the duration of treatment increases for patients with FASD. Again, a majority of the participants “Agree” that FASD increases treatment duration (53%, *n* = 42). However, others responded “Neither Disagree nor Agree” (28%, *n* = 22), “Strongly Agree” (16%, *n* = 13), “Disagree” (1%, *n* = 1), and “Strongly Disagree” (1%, *n* = 1). Third, the participants were asked if FASD adversely impacts the effectiveness of services provided. The most common responses were “Neither Disagree nor Agree” (48%, *n* = 38) and “Agree” (41%, *n* = 32). Very few participants endorsed either “Disagree” (6%, *n* = 5) or “Strongly Agree” (5%, *n* = 4). Fourth, the participants were asked how likely they would be to refer a client with FASD to an outside mental health provider. The most common responses were “I do not have clients with FASD” (29%, *n* = 23), “76% to 100%” (23%, *n* = 18), and “26% to 50%” (20%, *n* = 16). Less commonly endorsed options included “51% to 75%” (15%, *n* = 12) and “0% to 25%” (13%, *n* = 10).

Lastly, to identify the respondents’ preferred treatment strategies, the survey asked, “What interventions and strategies have you found most helpful when treating this population within the context of outpatient mental health treatment?” In this optional response, some respondents opted to identify specific interventions whereas others identified techniques and strategies. Interventions identified included cognitive behavioral therapy, behavioral therapy, family therapy, and applied behavioral analysis. Approaches and strategies identified included using concrete answers, emphasizing repetition, maintaining consistency and structure, working towards goals, developing social skills, and coordinating services. For a complete summary of responses, please see Table 1.

### 3.4. Suggestibility and Crime

To explore the less explored consequences of FASD, three additional questions were administered in relation to suggestibility and crime. First, the participants were asked if FASD increased an individual’s susceptibility to suggestion. The vast majority of respondents believed that FASD increased the risk of suggestibility (75%, *n* =59). Only 25% (*n* = 20) of participants responded that this was not the case. Further, the participants were asked if FASD increased an individual’s susceptibility to the unintentional creation of false memories (confabulation). Again, the majority of respondents believed that FASD increased the risk of confabulation (61%, *n* = 48). However, approximately 39% (*n* = 31) did not believe that FASD increased the risk of confabulation. Finally, the participants were also asked what percentage of individuals impacted by FASD become involved in the criminal justice system. The most common responses were that “26% to 50%” (37%, *n* = 39) and “51% to 75%” (30%, *n* = 24) of individuals with FASD become involved in the criminal justice system. Fewer respondents endorsed “1% to 10%” (16%, *n* = 13) or “11% to 25%” (16%, *n* = 13).

## 4. Discussion

FASD is a devastating condition that can be challenging for mental health professionals to deal with in clinical settings. There are FASD diagnostic clinics in many states. Additional information can be found at the websites of the National Organization on Fetal Alcohol Syndrome (NOFAS), which includes the Minnesota Organization on Fetal Alcohol Syndrome (MOFAS). The Minnesota Organization on Fetal Alcohol Syndrome recently renamed itself as Proof Alliance, additional information can be found at proofalliance.org. This study sought to better understand how professionals understood and serve clients with FASD. This study has four key findings. First, the majority of respondents (76%) had at least some FASD training within the last 5 years. Second, the vast majority of respondents could demonstrate basic knowledge of FASD. For example, respondents were able to identify key symptoms of FASD including deficits in cognitive function (e.g., executive function and attention), social skills, and adaptive functioning. Third, respondents typically recognized the impact of FASD of treatment length, effectiveness, and adherence. Fourth, the majority of respondents recognized FASD’s impact on suggestibility and crime. Together, these findings suggest that mental health professionals may be better equipped to understand and treat FASD than professionals over a decade ago. Nonetheless, future research is needed to better understand if and how these findings may generalize from the staff members of this facility to other settings across the United States.

### Limitations

This study had several limitations. Less than half the sampled population responded to complete the entire survey. Overall, participants totaled 79 individuals, which limited the power of the study. The participants that did complete the survey were overwhelmingly female with approximately four female participants for each male participant. Some of the research reported on crime involvement is somewhat dated and reinforces the need for more current studies in light of the changing legal landscape. The present study was unable to ascertain the sources participants utilized to obtain FASD training. Understanding where professionals are obtaining training on FASD would be substantially beneficial to the field’s efforts to increase knowledge about FASD in general.

## 5. Conclusions

FASD is a disorder which causes cognitive, social, and adaptive challenges throughout a person’s life. It is, therefore, imperative that mental health professionals remain cognizant of both the identification and treatment of clients with FASD. It must be recognized, however, that such identification is challenging due to misdiagnosis and undiagnosed situations. FASD does have a large scope of symptoms, some of which present physically and some without. There are often comorbid or secondary disabilities that affect the clients as well. Due to the executive and adaptive functioning deficits, assessment can be complicated with this population. Contributing to this, there is a lack of standardized and established FASD screening and assessment tools available to professionals. For these reasons, and more, mental health professionals require on-going training and education specific to working with individuals with FASD. This allows for continued growth in knowledge and understanding focused on the effects and impact of prenatal exposure to alcohol. With such awareness, individuals with FASD are more likely to receive support and servicing from mental health professionals.

## Figures and Tables

**Table 1:** Summary of responses.

Responses
Coordinated services
Black and white and concrete answers
Repetition
Impulse control related strategies such as: “Stop and think about the consequences before acting”
Narrative-based approaches
Increase social supports and services provided; repetitive and simplified Cognitive Behavioral Therapy (CBT) and interpersonal interventions; social skills training and practice (repetitive)
Client centered therapy
ABA therapy (Applied Behavioral Analysis)
Repetition of strategies, using visuals, parent education and support, connecting behavior to consequences and if it is a situation where we are discussing their behavior then discussing it without blame. For younger clients, token reinforcements seem to work well. For older clients, focusing on transition planning and ensuring continued support once they turn 18
Coordinating care with residential mental health support whenever possible (if client receives these services too, of course)
Depends on the client
Repetition, consistency, and mindfulness / coping skills
Behavioral interventions, helping them express their feelings, skills related work
Behavioral therapy
Consistency, clear and concrete expectations and communication with client and/or parent or guardian. Use of visuals definitely help
More in depth with questions and explanations. Working with the client on goals and treatment instead of choosing and having client adapt to my goals
Repetitive social skill training. one on one discussion, in order to gain insight into an issue, discuss something in multiple pieces, building on the information until the perspective is seen differently with empathy
SKILLS work, brain scanning and neurocognitive testing
Repetition, basic skills, Independent Living Skills (ILS)
Psych-education, slower pace of therapy, reducing own expectations, being flexible with strategies, exploring/developing support systems for client.
Emotional regulation through body relaxation and awareness. Social skills
CBT and skills-based supports

## Appendix A

### Survey Questions

1. Please describe the consequences associated with FASD? Choose all that apply.

2. Overall, what percentage of individuals (clients) impacted by FASD become involved in the criminal justice system? Choose one response.

3. Based on your knowledge, what percentage of individuals diagnosed with Fetal Alcohol Spectrum Disorder have facial abnormalities such as wideset eyes, thin upper lip, smooth philtrum, upturned nose, epicanthal folds, or small head size? Choose one response.

4. What are the most common co-occurring Mental Health Disorders amongst your clients with FASD? Choose all that apply.

5. Have you ever received training (education) related to FASD? Choose one response.

6. If you had training, how long ago was this training/education? Choose one response.

7. If you have received training on the recognition of FASD, how useful was it in helping you decide if someone has FASD? Choose one response.

8. Do you think having an FASD screening tool/app would help you screen more often for FASD? Choose one response.

9. How many years have you been working in the field of mental health? Choose one response.

10. In a typical month how many individuals do you interact with who are impacted by FASD as part of your duties as a mental health worker? Choose one response.

11. What % of your client’s with FASD also have a history of suicidal behaviors? Choose one response.

12. What % of your client’s with FASD also have (a) separate, one or more, co-occurring mental health disorder(s)? Choose one response.

13. What % of your client’s with FASD demonstrate impulse control issues? Choose one response.

14. FASD significantly impacts the accuracy of the data gathered from the patient during intake. Choose one response.

15. FASD significantly impacts the diagnostic data gathering process. Choose one response.

16. FASD plays a role in misdiagnosing of new patients. Choose one response.

17. FASD plays a role in patient compliance with keeping their scheduled mental health appointments. Choose one response.

18. FASD plays a role in patient adherence to a treatment plan. Choose one response.

19. The duration of treatment increases for patients with FASD. Choose one response.

20. FASD significantly impacts the social skills of the patient. Choose one response.

21. FASD significantly impairs the parenting ability of the patient. Choose one response.

22. FASD significantly adversely impacts the effectiveness of services provided. Choose one response.

23. How likely are you to refer a patient with FASD to an outside mental health provider? Choose one response.

24. Do you believe FASD increases an individual’s susceptibility to suggestion? Choose one response.

25. Do you believe FASD increases an individual’s susceptibility to the unintentional creation of false memories (confabulation)? Choose one response.

26. What interventions and strategies have you found most helpful when treating this population within the context of outpatient mental health treatment? Answering is optional.

27. What is your gender? Choose one response.

28. What is your age range? Choose one response.

29. Do you feel education/training in the identification of FASD should occur regularly? Choose one response.

30. Would you benefit from a continuing education course that addresses the interaction between FASD and the mental health system? Choose one response.

31. What is your highest completed level of education? Choose one response.

32. What is your role at Headway? Please list your title and a brief job description. Answering is optional.
